# Antibacterial Activity and Bonding Ability of an Orthodontic Adhesive Containing the Antibacterial Monomer 2-Methacryloxylethyl Hexadecyl Methyl Ammonium Bromide

**DOI:** 10.1038/srep41787

**Published:** 2017-02-07

**Authors:** Fan Yu, Yan Dong, Hao-han Yu, Ping-ting Lin, Ling Zhang, Xiang Sun, Yan Liu, Yu-ning Xia, Li Huang, Ji-hua Chen

**Affiliations:** 1 State Key Laboratory of Military Stomatology & National Clinical Research Centre for Oral Diseases & Shaanxi Key Laboratory of Oral Diseases, Department of Prosthodontics, School of Stomatology, Fourth Military Medical University, Xi’an, China; 2 State Key Laboratory of Military Stomatology & National Clinical Research Centre for Oral Diseases & Shaanxi Engineering Research Center for Dental Materials and Advanced Manufacture, Department of VIP Dental Care, School of Stomatology, Fourth Military Medical University, Xi’an, China; 3 State Key Laboratory of Military Stomatology & National Clinical Research Center for Oral Diseases & Shaanxi International Joint Research Center for Oral Diseases, Department of General Dentistry and Emergency, School of Stomatology, Fourth Military Medical University, Xi’an, Shaanxi, China

## Abstract

Irreversible white spot lesion (WSL) occurs in up to 50% of patients during orthodontic treatment. Therefore, orthodontic adhesives need to be able to inhibit or reduce bacterial growth in order to prevent or minimize WSL. This study evaluated the antibacterial effect and shear bond strength (SBS) of a resin-based orthodontic adhesive containing the antibacterial monomer 2-methacryloxylethyl hexadecyl methyl ammonium bromide (MAE-HB). MAE-HB was added at three concentrations (1, 3, and 5 wt%) to a commercial orthodontic adhesive Transbond XT, while the blank control comprised unmodified Transbond XT. Their antibacterial effects on *Streptococcus mutans* were investigated after 0 and 180 days of aging. The SBS of metal brackets bonded to the buccal enamel surface of human premolars was assessed. Compared with the blank control, the MAE-HB-incorporated adhesive exhibited a significant contact inhibitory effect on the growth of *S. mutans* (P < 0.05), even after 180 days of aging. SBS and adhesive remnant index values revealed that the bonding ability of the experimental adhesive was not significantly adversely affected by the incorporation of MAE-HB at any of the three concentrations. Therefore, orthodontic adhesives with strong and long-lasting bacteriostatic properties can be created through the incorporation of MAE-HB without negatively influencing bonding ability.

Orthodontic treatment usually fails to meet the aesthetic and functional health expectations of both the patients and clinicians. This is due to the most common side effects of enamel demineralization or white spot lesion (WSL) formation around orthodontic brackets^1^,^2^,^3^,^4^. There is also an increased prevalence of cariogenic streptococci in the dental biofilm surrounding the brackets^5^,^6^ and the occurrence of unaesthetic, unhealthy, and potentially irreversible WSL is found in up to 50% of patients during orthodontic treatment^7^,^8^,^9^,^10^. Several methods have been introduced in an attempt to meet clinical requirements, such as fluoride varnishes, fluoride mouth rinses, oral hygiene instructions, and the development of other antibacterial orthodontic materials^6^,^11^,^12^,^13^,^14^.

Resin-based orthodontic adhesives have been used for the bonding of the brackets to the enamel; however, they can facilitate high levels of microorganism adhesion owing to their rough surface that provides ideal sites for the rapid attachment and growth of oral microorganisms^15^,^16^. Larger amounts of bacteria have been detected on the adhesive than on the bracket material itself^16^. Sukontapatipark *et al*.^17^ reported bacterial accumulation within 10-μm wide gaps at the adhesive-enamel junction around the bracket base. Therefore, adhesives with antimicrobial properties or microbial repellent actions are desirable^6^,^18^,^19^,^20^.

Several investigations have been performed to endow adhesives with antibacterial properties. Owing to their effective antibacterial properties, chlorhexidine and fluorine-containing agents have been previously mixed with adhesives; however, the release of the active component limits the duration of their effect and may influence the mechanical characteristics of the adhesive^21^,^22^. Furthermore, other Ag-loaded antibacterial materials have been shown to be limited due to discoloration and reduced esthetics^23^,^24^. To address these problems, polymerizable quaternary ammonium salt (QAS) monomers have been introduced to endow a dental material with antibacterial activity, including 12-methacryloyloxydodecylpyridinium bromide (MDPB) and methacryloxylethyl cetyl dimethyl ammonium chloride (DMAE-CB). Incorporation of these monomers into adhesives have been shown to cause reliable reduction in bacterial growth before and after curing^25^,^26^. However, a MDPB monomer had some limitations. Since only one aliphatic C = C bond occurs in its molecular structure, the amount of MDPB incorporated into the resin matrix is limited and this may lead to poor reproducibility of anti-plaque effects. Indeed, when it has been used to bond brackets to enamel surfaces, reduction in bond strength has been reported^27^.

In a previous study, we synthesized a novel QAS monomer, 2-methacryloxylethyl hexadecyl methyl ammonium bromide (MAE-HB)^28^ that has a strong bactericidal activity against *Streptococcus mutans, Actinomyces viscosus, Lactobacillus acidophilus, Staphylococcus aureus, Streptococcus sanguinis, Porphyromonas gingivalis, Prevotella melaninogenica*, and *Enterococcus faecalis*. In contrast with MDPB, it contains two aliphatic C = C bonds, providing considerably more active surfaces with higher densities of immobilized antimicrobial agents and consequently greatly improving the antibacterial activities of the resin material^29^. However, its antibacterial activity when copolymerized with resin matrix is not yet known. A variety of factors such as microbes, surfaces, and the surrounding environments may be involved in the antibacterial action^30^. The physicochemical properties of the material may be changed due to the variation of the components, thus will exert an influence on the antibacterial activity^30^,^31^,^32^,^33^. We therefore incorporated MAE-HB into a commercially orthodontic adhesive to assess its effect on the inhibition on WSL. The objectives of this study were to investigate the *in vitro* antibacterial activity of the MAE-HB-incorporated adhesive and to evaluate its influence on the bonding ability of the adhesive after curing.

## Results

### *Streptococcus mutans* biofilm growth on material surfaces with or without aging

**Figure 1:**
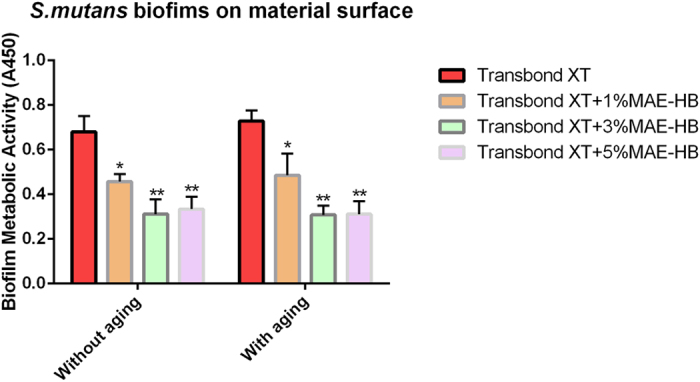
Metabolic activity of *S. mutans* biofilms adhered to Transbond XT, Transbond XT+1%MAE-HB, Transbond XT+3%MAE-HB, and Transbond XT+5%MAE-HB. Metabolic activity was measured via the CCK-8 assay at 1d and after aging for 6 months. In each plot, values (mean ± standard deviation; n = 6) with dissimilar letters are significantly different (P < 0.05).

The colony-forming-unit (CFU) counts of *S. mutans* on the surfaces of the tested materials with or without aging are shown in Table 1. Two-way ANOVA showed that only material type had significant effect on the CFU count (P < 0.05). For each group, aging treatment did not affect the CFU counts (P > 0.05). The CFU counts in the Transbond XT+1%MAE-HB group were significantly lower, by approximately an order of one magnitude, than that for the Transbond XT group (P < 0.001). No significant difference was observed between the Transbond XT+3%MAE-HB and Transbond XT+5%MAE-HB groups (P > 0.05). Figure 1 shows the metabolic activity of *S. mutans* on the surfaces of the tested materials with or without aging. The results were in accordance with the CFU counts test. Compared with Transbond XT, bacterial metabolic activities were significantly suppressed on 1, 3, and 5 wt%-MAE-HB-incorporated adhesives (P < 0.05).

### *Streptococcus mutans* biofilm growth in culture medium according to different aging conditions

**Figure 2:**
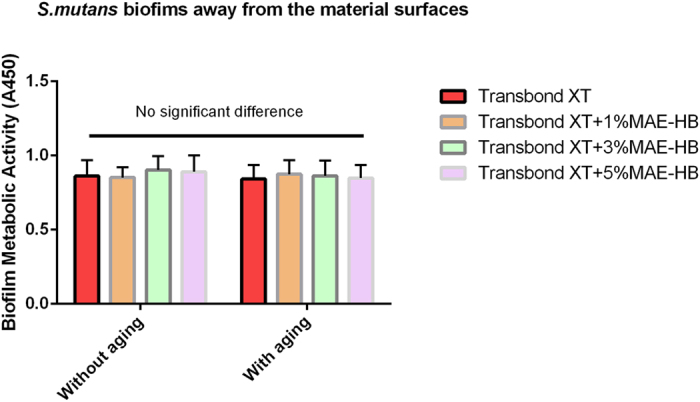
Metabolic activity of *S. mutans* biofilms growth in culture medium. Metabolic activity was measured via the CCK-8 assay at 1 d and after aging for 6 months. In each plot, values (mean ± standard deviation; n = 6) with dissimilar letters are significantly different (P < 0.05).

Table 2 lists the CFU counts of *S. mutans* from the culture medium according to different aging conditions. Two-way ANOVA showed that both material type and aging had no significant effect on the CFU count (all P > 0.05). In addition, one-way ANOVA revealed no significant differences between all subgroups with different aging conditions (all P > 0.05). Figure 2 shows the metabolic activity of *S. mutans* from the culture medium according to different aging conditions. The results were in line with the CFU counts test. There was no significant difference observed between the different groups (P > 0.05).

### Confocal laser scanning microscopy analysis

**Figure 3:**
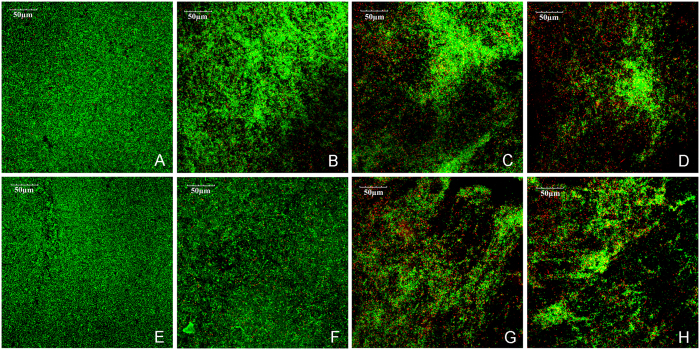
LIVE/DEAD fluorescence images of *S. mutans* biofilms on material surfaces with or without aging. Representative CLSM images of LIVE/DEAD stained *S. mutans* biofilms after 24 h of anaerobic growth on the tested material surfaces: (**A**) Transbond XT, (**B**) Transbond XT+1%MAE-HB, and, (**C**) Transbond XT+3%MAE-HB, (**D**) Transbond XT+5%MAE-HB. *Streptococcus mutans* biofilms on the corresponding aged samples are shown in **E–H**. Live bacteria were stained green, and the compromised bacteria were stained red. When the live and dead bacteria are close to each other or on the top of each other, the yellowish or orange fluorescence is presented. Scale bars, 500 μm.

Figure 3 shows representative LIVE/DEAD staining images of *S. mutans* biofilms on material surfaces with or without aging. Live bacteria were stained green and the compromised bacteria were stained red. When the live and dead bacteria were in close proximity, the biofilm was co-stained with the two fluorophores, resulting in yellowish or orange colors. For each material, the LIVE/DEAD staining results were qualitatively similar between the fresh and aged surfaces. The biofilms on Transbond XT were predominantly viable, with small amounts of dead cells (Fig. 3A,E). There was a slight increase in the amount of dead cells on Transbond XT+1%MAE-HB (Fig. 3B,F). Compared with Transbond XT, there was noticeably less green staining, and more red/orange staining in the biofilms both on Transbond XT+3%MAE-HB and Transbond XT+5%MAE-HB (Fig. 3C,D,G,H).

### Fe-SEM observation

**Figure 4:**
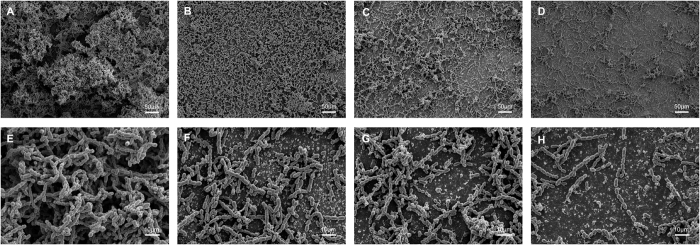
Fe-SEM images of *S. mutans* accumulation and morphology after anaerobic inoculation. Accumulation after anaerobic inoculation with (**A**) Transbond XT, (**B**) Transbond XT+1%MAE-HB, and, (**C**) Transbond XT+3%MAE-HB, (**D**) Transbond XT+5%MAE-HB. Morphology after anaerobic inoculation with (**E**) Transbond XT, (**F**) Transbond XT+1%MAE-HB, and, (**G**) Transbond XT+3%MAE-HB, (**H**) Transbond XT+5%MAE-HB.

Figure 4 shows representative Fe-SEM images of *S. mutans* biofilms on material surfaces after 4 h of incubation. The Fe-SEM observations showed that, after anaerobic growth at 37 °C for 4 h, a significant amount of *S. mutans* accumulated on the surface of Transbond XT with normal morphology (Fig. 4A,E). In contrast, a reduced amount of *S. mutans* was found on the surface of MAE-HB modified adhesives (Fig. 4B–D). The amount of *S. mutans* on the Transbond XT+5%MAE-HB appeared to be the lowest. High magnification images revealed a normal morphology of *S. mutans* on 0%-MAE-HB modified Transbond XT (Fig. 4E), some cell debris were also found on the surfaces of the MAE-HB modified adhesives (Fig. 4F,G,H).

### Agar diffusion assay

After 48 h of agar diffusion assay, no inhibitory halos were detected around disks of any experimental adhesives (Supplementary Figure S1).

### Bonding property

Shear bond strength (SBS) values (expressed in MPa) are shown in Table 3. There were no significant differences between the three experiment materials and the control group (P > 0.05). The SBS values were all approximately 10 MPa. The ARI scores, indicating the amount of adhesive remaining after the SBS test, are shown in Table 4. ARI scores in the four groups were mainly distributed within the range of 1 and 2. There was no significant difference between groups (P > 0.05).

## Discussion

Direct bracket bonding to the etched enamel surface has simplified orthodontic treatment operation and improved esthetic outcomes; however, it also has several disadvantages^34^. The main problems are surface enamel loss and demineralization near the bracket^35^. Materials with antibacterial or demineralizing properties are required to improve prognosis. Therefore, orthodontic adhesives need to be able to inhibit or reduce bacterial growth in order to prevent or minimize WSL^36^. A considerable amount of research has been performed to eliminate bacterial-induced WSL during orthodontic treatment.

In recent years, extensive research has been performed on the development of antibacterial restorative materials. These materials can be divided into two types: agent-releasing antibacterial materials and non-agent-releasing antibacterial materials. Agent-releasing antibacterial adhesives contain antibacterial components such as fluorine and chlorhexidine; However, tight control of the release kinetics at a durable minimum inhibitory concentration of antimicrobials remains a challenge; the functional agents release will decrease with time^37^ and may be insufficient for a maximal antibacterial effect^38^, thereby rendering dental materials with limited antibacterial activities^14^,^39^,^40^,^41^. Moreover, the release of the agent may adversely influence the physical properties of the carrier material, and can lead to undesirable side effects such as tooth discoloration and underlying cytoxicity^41^,^42^,^43^. In contrast, QAS (MDPB for example) is a cationic active biocide that can be copolymerized with a resin matrix and exhibit marked antimicrobial activity against a wide range of bacteria, fungi, and viruses^28^,^44^. Therefore, in this study, MAE-HB was used as the antibacterial agent for copolymerization with an adhesive and its antibacterial activity and bonding properties were evaluated *in vitro*.

*Streptococcus mutans* is a Gram-positive bacteria that resides in multispecies biofilms on the surfaces of the teeth^45^. Oral colonization by *S. mutans* has been shown to be elevated around the brackets and on the surface of the existing adhesives^36^. Therefore, we aimed to evaluate the antibacterial effects of an adhesive containing MAE-HB. Our findings relating to *S. mutans* growth and metabolic activity consistently demonstrated that the addition of MAE-HB into the adhesive resulted in a significant reduction in bacterial growth, under different aging conditions, compared with the control group. In addition, none of the materials affected bacterial growth or metabolic activity in the solution surrounding the samples. This was also confirmed by the agar diffusion assay; none of the modified adhesives showed an inhibition zone around the disks, indicating that no antibacterial agents were released from the adhesives. This indicates that MAE-HB was completely immobilized into the parent material and does not leach out over time, thereby providing a durable contact-antibacterial capability^43^ and minimizing cytotoxicity^46^.

The adhesives with 3 and 5 wt% MAE-HB showed a more effective long-term anti-*S. mutans* activity than the 1 wt% MAE-HB group. The results of LIVE/DEAD staining and Fe-SEM were consistent with those for metabolic activity and CFU formation. We recorded lower amounts of live bacteria adhered to the MAE-HB-incorporated adhesive and membrane integrity was reduced to a lower extent compared with the control. The Fe-SEM images showed a destructive bacteria morphology on the surfaces of MAE-HB modified adhesives. Our findings are comparable to those of previous investigations of cured MDPB and DMAE-CB-incorporated adhesives that showed a similar inhibition on the growth of *S. mutans*^43^,^47^. The present study demonstrates that Transbond XT alone did not show effective antibacterial activity upon contact; large amounts of bacteria were found on its rough surface. The agar diffusion assay also indicated that Transbond XT do not possess antibacterial activity. Previous studies also reported the limited antibacterial ability of Transbond XT^41^,^48^. The reason for this may be that it does not contain fluorine or any other antibacterial reagents.

As cationic agents, QAS monomers are assumed to exert biocide activity by reacting with a negatively charged bacterial surface, causing membrane damage and irreversible loss of cytoplasmic constituents^46^,^49^. Our previous study showed that QAS monomers are able to attenuate the *gtfB* gene expression of *S. mutans*^50^, thereby decreasing glucan synthesis and subsequently inhibiting bacterial adhesion and the generation of caries^45^. QAS monomers are also reported to have anti-matrix metalloproteinase (MMP) activity^51^; when MMP in bacteria are inhibited, microbial cell homeostasis is disrupted and growth is retarded^52^,^53^. In the present study, MAE-HB was chemically bound in the matrix of the adhesive after curing and could therefore exhibit a bacteriostatic effect upon contact.

Resin-based material may experience a mechanical property loss when different antibacterial agents are added. It is highly recommended that orthodontic adhesives should possess an appropriate bonding strength, ranging between 5.9 and 7.8 MPa, to permit adequate adhesion and facilitate debonding^51^,^54^. In the current research, the SBS values of all groups were approximately 10 MPa. Therefore, adhesives with 1, 3, and 5 wt% MAE-HB incorporation may provide sufficient bonding strength comparable to the commercial adhesive Transbond XT, which is viewed as the orthodontic gold standard adhesive^55^. After debonding, adhesives remaining on the enamel surface around the bracket base may provide a rough surface for bacterial colonization. Following bracket removal, residual adhesive remaining on the enamel surface should be reduced as much as possible to meet clinicians’ primary goal^56^,^57^. According to our results, there was no significant difference in ARI score between adhesives containing 0, 1, 3, and 5 wt% MAE-HB. Based on the SBS and ARI findings, the MAE-HB-incorporated adhesive has a clinically relevant range of bonding properties similar to that of a commercial adhesive. However, considering potential occlusion stress, the current SBS experiment may have not accurately reflected the clinical situation. Therefore, the actual bonding property of this adhesive based on clinical observation needs to be determined.

Resin-based adhesives provide several advantages such as elimination of pretreatment, decreased gingival irritation, easier oral hygiene, improved esthetics, and reduced chair side time. However, they are also associated with high levels of bacterial adhesion. In this study, the incorporation of MAE-HB into the commercial adhesive Transbond XT showed a contact-inhibition activity against *S. mutans* and the antibacterial effect was maintained even after water-aging for 6 months. The bonding strength of the parent material was not attenuated by the incorporation of MAE-HB, suggesting that the developed material has potential application as an antibacterial adhesive in orthodontic treatment, while its bonding durability *in vivo* and *in vitro* requires further study. Incorporation of QAS monomers into orthodontic adhesives may be an effective strategy for achieving long-term antibacterial efficacy. However, further studies are required to investigate the antibacterial properties and enamel bond strength of adhesives containing MAE-HB *in-vivo*, as well as the mechanical characteristics.

## Methods

### Materials

**Figure 5:**
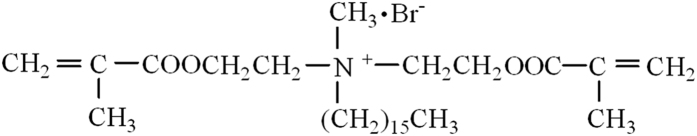
Structure of 2-methacryloxylethyl hexadecyl methyl ammonium bromide.

The structure of MAE-HB is presented in Fig. 5. A commercially available orthodontic adhesive, Transbond XT (3 M Unitek, Monrovia, CA) was used as the parent material for antibacterial functionalization. For the test material, we used Transbond XT supplemented with 1, 3, and 5 wt% QAS monomer MAE-HB. Transbond XT without the MAE-HB monomer served as the negative control.

### Antibacterial testing

#### Sample preparation

MAE-HB 1, 3, and 5 wt% were pre-incorporated into Transbond XT adhesive paste. Plates containing 24 wells (Costar, Corning, Lowell, MA, USA) were used as a mold for specimen preparation. Fifty microliters of each test material were spread evenly on the bottom of the 24-well plate (six samples for each concentration group) and light cured for 20 seconds with a curing unit (Dentsply QHL 75, Milford, DE, USA). The diameter of each specimen was approximately 8 mm. All specimens were dried at room temperature, sterilized with ethylene oxide gas, and degassed for more than 48 h.

For the aging progress, six specimens of each group were placed in a 24-well plate containing 1 ml distilled water that was replenished every 3 days. After aging for 6 months at 37 °C, specimens were retrieved, sterilized, and subjected to the following tests.

#### Bacterial strains and culture conditions

*Streptococcus mutans* UA159 was provided by the Endodontics Department and Microbiology Department of the Fourth Military Medical University and cultured overnight at 37 °C in brain heart infusion (BHI) broth (Difco, Detroit, MI, USA) in an anaerobic atmosphere (90% N_2_, 5% CO_2_, and 5% H_2_). Bacterial suspension was adjusted to 1 × 105 colony-forming units (CFU)/mL for further use.

#### Bacterial growth on material surfaces and in culture medium, before and after aging process

Twenty microliters of the diluted *S. mutans* suspension was added to the surface of each prepared disk with or without aging supplement in a 2 ml BHI broth. After anaerobic culture for 24 h, *S. mutans* growth on the disk and planktonic bacteria in the culture medium were assessed via CFU counts and metabolic testing as previous described^58^.

After 24 h in culture to allow biofilm growth, disks were washed twice with phosphate-buffered saline and then transferred into 15-ml sterile centrifuge tubes with 2 ml fresh BHI. Biofilms growing on material surfaces were collected by sonication (3510R, Branson, Danbury, CT, USA) for 3 min and vortex mixing at a maximum speed of 20 s using a vortex mixer (Fisher Scientific, Pittsburgh, PA, USA). Once the disks had been removed from the wells for biofilm harvesting, planktonic bacteria in the original medium samples were mixed thoroughly by repeated pipetting to achieve a homogeneous bacterial suspension.

For the CFU counts, obtained bacterial suspensions from both the biofilms on the disks and the planktonic bacteria in the medium were serially diluted and inoculated on BHI agar plate for 1 day at 5% CO_2_ and 37 °C to determine the total number of CFU recovered.

For metabolic testing, a Cell Counting Kit-8 (7Sea Biotech, Shanghai, China) was used according to the manufacturer’s instructions. Briefly, 200 μl of obtained bacterial suspensions from each group were transferred into a 96-well plate, and then 20 μl of Cell Counting Kit-8 (CCK-8) dye solution was added to each well. After incubation at 37 °C in 5% CO_2_ for 2 h, the absorbance at 450 nm was measured using a microplate reader (SpectraMax M5, Molecular Devices, Sunnyvale, CA, USA). Each sample was assayed in triplicate, and an average value was calculated for each sample.

#### Confocal laser scanning microscopy analysis

After 24 h incubation, *S. mutans* adherent on each sample with or without aging were analyzed by confocal laser scanning microscopy (CLSM; FluoView FV1000, Olympus, Tokyo, Japan). The disks coated with biofilms were washed three times with sterile saline to remove loose bacteria. The fluorescent LIVE/DEAD BacLight Bacterial Viability stain (LIVE/DEAD BacLightTM Bacterial Viability Kit L7012, Molecular Probes, Inc., Eugene, OR, USA) containing SYTO 9 and propidium iodide was used for the staining of a total of 36 specimens according to the manufacturer’s instructions. Following this, the samples were rinsed gently with distilled water and observed under CLSM (FluoView FV1000, Olympus). Excitation with a 488-nm laser revealed the green fluorescence emission of live bacteria, and excitation with a 543-nm laser revealed the red fluorescence emission of bacteria with damaged membranes.

#### Field emission scanning electron microscope observation

After a 24-h incubation, *S. mutans* adherent on each sample without aging were analyzed by field emission scanning electron microscope (Fe-SEM, S-4800, Hitachi, Tokyo, Japan). Briefly, the specimens with biofilm were gently rinsed with distilled water and fixed in 2.5% glutaraldehyde in 0.1 mol/l cacodylate buffer at pH 7.2 for 4 h at 37 °C. Specimens were then dehydrated in an ascending ethanol series with a critical-point drier. After being coated with gold using an ion sputter (JFC-1100E, JEOL, Japan), the central portion of the specimens was observed with Fe-SEM.

#### Agar diffusion assay

Samples were prepared as described above using a 96-well plate. Agar diffusion test were performed according to a previous study^48^. Four disks of each group were placed on agar plates with 150 μL of grown *S. mutans* spread with glass balls. After anaerobic culture for 48 h, the plates were visually inspected for the presence of inhibitory zones in the bacterial coat. The inhibitory halo of each disk was measured in millimeters using a manual caliper. This was repeated three times, and mean values were used for analysis.

### Bonding property test

For the adhesive property test, 40 freshly extracted human premolars without caries or fractures were investigated. The teeth were stored in distilled water at 4 °C and thymol crystals 0.2% (wt/vol) were added to inhibit bacterial growth. The 40 teeth were randomized into four equal groups. Stainless steel premolar brackets (Xinya dental material, Hangzhou, China) with a base area of 12.97 mm^2^ (obtained from the manufacturer) were used in this study.

Before the bonding process, the buccal enamel surface of each tooth was cleansed, polished with fluoride-free paste for 15-second, and thoroughly air-dried. For each group, the buccal surface was etched with 37% phosphoric acid gel for 30 seconds, rinsed with water for 30 seconds, and dried with oil- and moisture-free air until a frosty white appearance was achieved. Following this, the Transbond XT primer was applied to the etched surface in a thin uniform film and photo activated for 10 seconds with a light activation unit (QHL75; Dentsply, Tulsa, OK, USA). Transbond XT adhesive paste with or without MAE-HB was applied to the bracket base, and the bracket was positioned on the tooth. Excess adhesive around the bracket base was removed, and the adhesive was light-cured for 10 seconds on each side of the bracket. Each premolar was individually fixed in acrylic resin to ensure that the facial surface of the tooth was parallel to the applied force during the shear bond test.

After 24 hours of storage in distilled water at 37 °C, the specimens were submitted to a shear bond strength (SBS) test using a universal testing machine. An occlusal-gingival load was applied to produce a shear force at the bracket-tooth interface. The SBS was measured at a crosshead speed of 0.5 mm per minute, and the load at the time of debonding was recorded in megapascals (MPa).

After debonding, the amount of residual adhesive adhering to the enamel surface was quantified by using the adhesive remnant index (ARI) developed by Årtun and Bergland^53^. The ARI scores of all samples were recorded using an optical stereomicroscope under 10 × magnification. Scoring groups were as follows: 0, no adhesive remains on the tooth; 1, less than 50% of the adhesive remains on the tooth; 2, more than 50% of the adhesive remains on the tooth; and 3, all adhesive remains on the tooth.

### Statistical analysis

Two-way analyses of variance (ANOVA) was performed to detect the significant effects of the variables (material type and aging status) on CFU count and bacterial metabolic activity, while Tamhane multiple comparison test was used to compare differences between any two groups. One-way ANOVA and least significant difference post-hoc tests were used to determine differences in SBS values among groups. The chi-square test was used to evaluate the ARI scores. Statistical significance for all statistical tests was predetermined at P = 0.05.

## Additional Information

**How to cite this article**: Yu, F. *et al*. Antibacterial Activity and Bonding Ability of an Orthodontic Adhesive Containing the Antibacterial Monomer 2-Methacryloxylethyl Hexadecyl Methyl Ammonium Bromide. *Sci. Rep.* **7**, 41787; doi: 10.1038/srep41787 (2017).

**Publisher's note:** Springer Nature remains neutral with regard to jurisdictional claims in published maps and institutional affiliations.

## Supplementary Material

Supplementary Figure S1

## Figures and Tables

**Table 1:** Mean (standard deviation) colony-forming unit (CFU) counts of *S. mutans* biofilms on material surfaces.

Groups	CFU	
	Without aging	With aging
Transbond XT	6.14 (0.77) × 10^8A^	6.48 (0.44) × 10^8A^
Transbond XT+1%MAE-HB	7.18 (1.04) × 10^7B^	6.52 (0.70) × 10^7B^
Transbond XT+3%MAE-HB	5.34 (0.67) × 10^6C^	5.53 (0.68) × 10^6C^
Transbond XT+5%MAE-HB	5.20 (0.42) × 10^6C^	5.44 (0.56) × 10^6C^

**Table 2:** Mean (standard deviation) colony-forming unit (CFU) counts in *S. mutans* biofilms from the culture medium according to different aging conditions.

Groups	CFU	
	Without aging	With aging
Transbond XT	1.44 (0.16) × 10^9A^	1.46 (0.13) × 10^9A^
Transbond XT+1%MAE-HB	1.42 (0.17) × 10^9A^	1.47 (0.18) × 10^9A^
Transbond XT+3%MAE-HB	1.49 (0.13) × 10^9A^	1.44 (0.12) × 10^9A^
Transbond XT+5%MAE-HB	1.44 (0.13) × 10^9A^	1.49 (0.16) × 10^9A^

**Table 3:** Shear bond strength (SBS) values of the orthodontic adhesives (MPa).

Group	Number	SBS			
		Mean	Standard deviation	Min	Max
Transbond XT	10	10.31	2.22	6.97	13.32
Transbond XT+1%MAE-HB	10	10.33	2.42	6.12	14.03
Transbond XT+3%MAE-HB	10	10.26	2.55	6.99	14.18
Transbond XT+5%MAE-HB	10	10.2	2.7	6.45	14.05

**Table 4:** Adhesive remnant index (ARI) scores of orthodontic adhesives.

Groups	ARI scores			
	0	1	2	3
Transbond XT	0	5	4	1
Transbond XT+1%MAE-HB	0	4	5	1
Transbond XT+3%MAE-HB	1	5	4	0
Transbond XT+5%MAE-HB	0	4	4	2
