# Supplementary figures and images for: Antibacterial Activity and Bonding Ability of an Orthodontic Adhesive Containing the Antibacterial Monomer 2-Methacryloxylethyl Hexadecyl Methyl Ammonium Bromide
Source: Sci Rep. 2017 Feb 7;7:41787. doi: 10.1038/srep41787 (PMC5294631; doi:10.1038/srep41787)

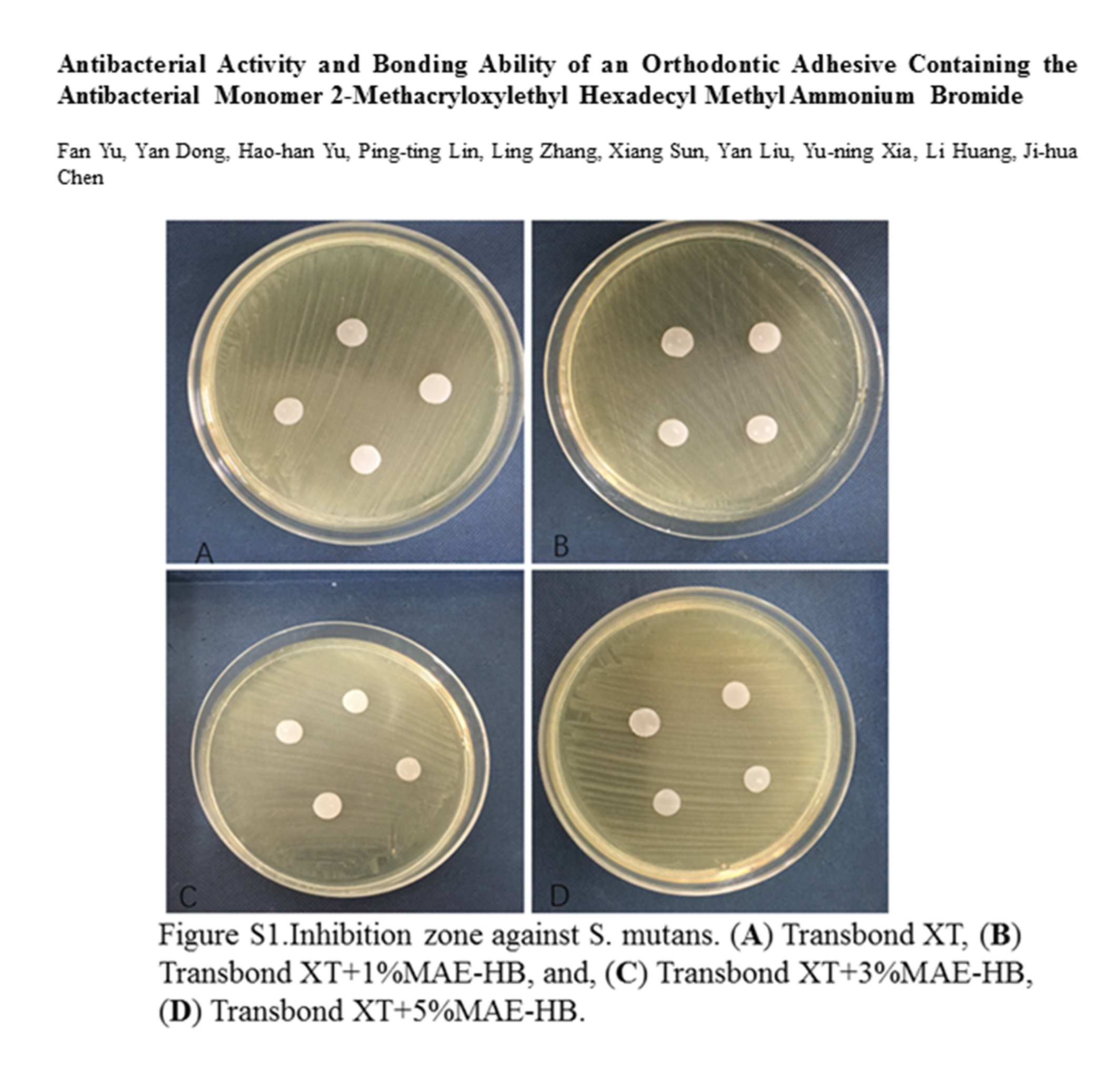

Supplement: Supplementary Figure S1 [file srep41787-s1.tiff]
